# Resistance-Associated Mutations in Chronic Lymphocytic Leukemia Patients Treated With Novel Agents

**DOI:** 10.3389/fonc.2020.00894

**Published:** 2020-06-25

**Authors:** Lenka Sedlarikova, Anna Petrackova, Tomas Papajik, Peter Turcsanyi, Eva Kriegova

**Affiliations:** ^1^Department of Immunology, Faculty of Medicine and Dentistry, Palacky University and University Hospital, Olomouc, Czechia; ^2^Department of Hemato-Oncology, Faculty of Medicine and Dentistry, Palacky University and University Hospital, Olomouc, Czechia

**Keywords:** CLL, targeted therapy, resistance-associated mutations, treatment resistance, genetic aberrations

## Abstract

Inhibitors of B-cell receptor signaling, ibrutinib and idelalisib, and BCL-2 antagonist, venetoclax, have become the mainstay of treatment for chronic lymphocytic leukemia (CLL). Despite significant efficacy in most CLL patients, some patients develop resistance to these agents and progress on these drugs. We provide a state-of-the-art overview of the acquired resistance to novel agents. In 80% of patients with ibrutinib failure, acquired mutations in *BTK* and *PLCG2* genes were detected. No distinct unifying resistance-associated mutations or deregulated signaling pathways have been reported in idelalisib failure. Acquired mutations in the *BCL2* gene were detected in patients who had failed on venetoclax. In most cases, patients who have progressed on ibrutinib and venetoclax experience resistance-associated mutations, often present at low allelic frequencies. Resistance-associated mutations tend to occur between the second and fourth years of treatment and may already be detected several months before clinical relapse. We also discuss the development of next-generation agents for CLL patients who have acquired resistant mutations to current inhibitors.

## Introduction

In the last decade, the great shift in the therapeutic management of chronic lymphocytic leukemia (CLL) is attributed to the approval of two B-cell receptor (BCR) signaling pathway inhibitors (ibrutinib and idelalisib) and the BCL-2 antagonist venetoclax. These novel agents have shown significant clinical efficacy in high-risk patients with relapsed/refractory (R/R) disease with 17p deletion and/or *TP53* mutation and complex karyotype as well as in previously untreated patients with/without poor-risk features (1–4). Despite the induction of long-term remission in most CLL patients, in some patients, the treatment fails. The number of patients who progress or develop clinical resistance is expected to increase with the growing number of patients indicated for this treatment and due to the long-term administration of these agents. Therefore, understanding the mechanisms driving resistance and identification of involved driver mutations and signaling pathways is a current need. These findings will help to design new targeted therapies to overcome resistance and to find drug combinations that will prevent the development of resistance or will help to avoid relapse. This manuscript provides an up-to-date overview of the acquired resistance-associated mutations in CLL patients treated with ibrutinib, idelalisib, and venetoclax. These mutations emerge during the treatment course and predispose to the loss of function of the drug and disease progression. Moreover, we summarize recent findings in ways on how to manage the patients who have acquired resistant mutations to current inhibitors.

## Resistance-Associated Mutations in Ibrutinib Treatment

**Figure 1:**
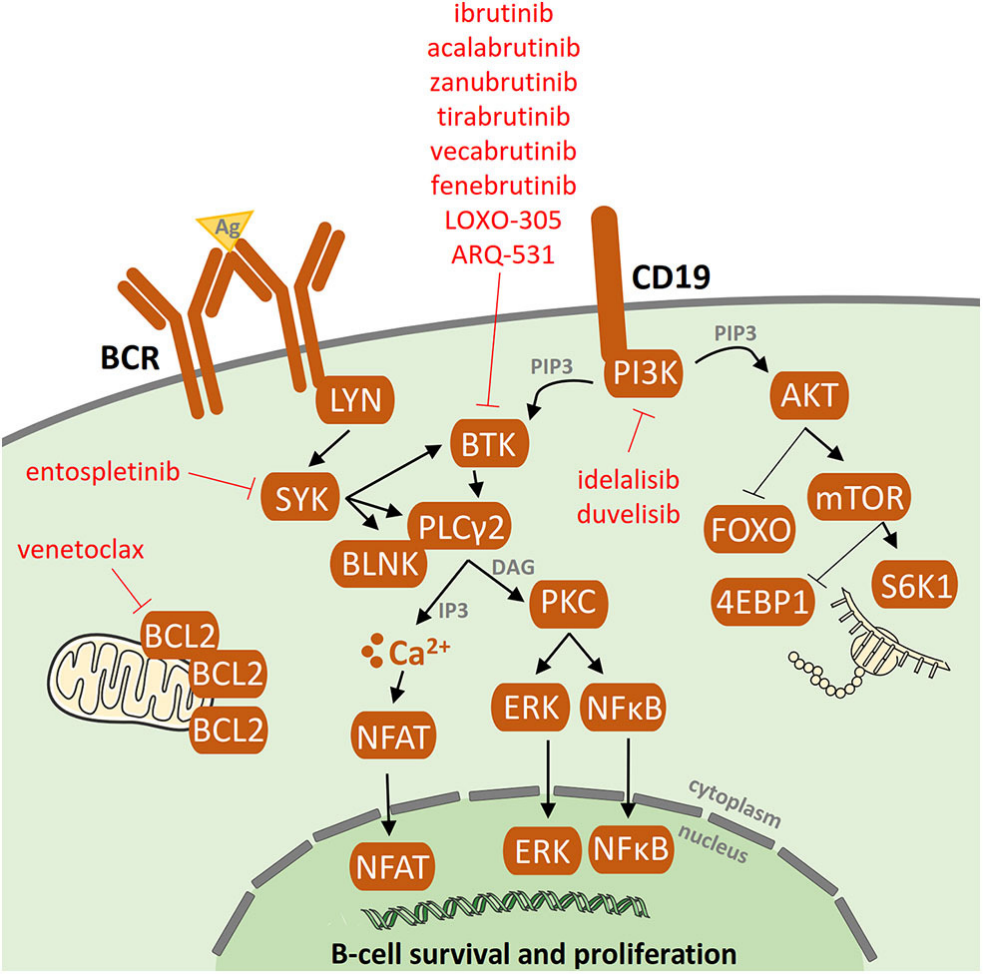
B-cell receptor (BCR) signaling pathway and BCR inhibitors. Following antigen binding to the B-cell receptors, BCR signaling is chronically activated in CLL cells. As a consequence, the activated BCR pathway results in the production of second messengers and the activation of NF-kB and subsequent pro-proliferation and antiapoptotic pathways. Targets of the BCR inhibitors: ibrutinib, idelalisib, acalabrutinib, zanubrutinib, tirabrutinib, fenebrutinib, vecabrutinib, LOXO-305, ARQ-531, and entospletinib are depicted.

**Figure 2:**
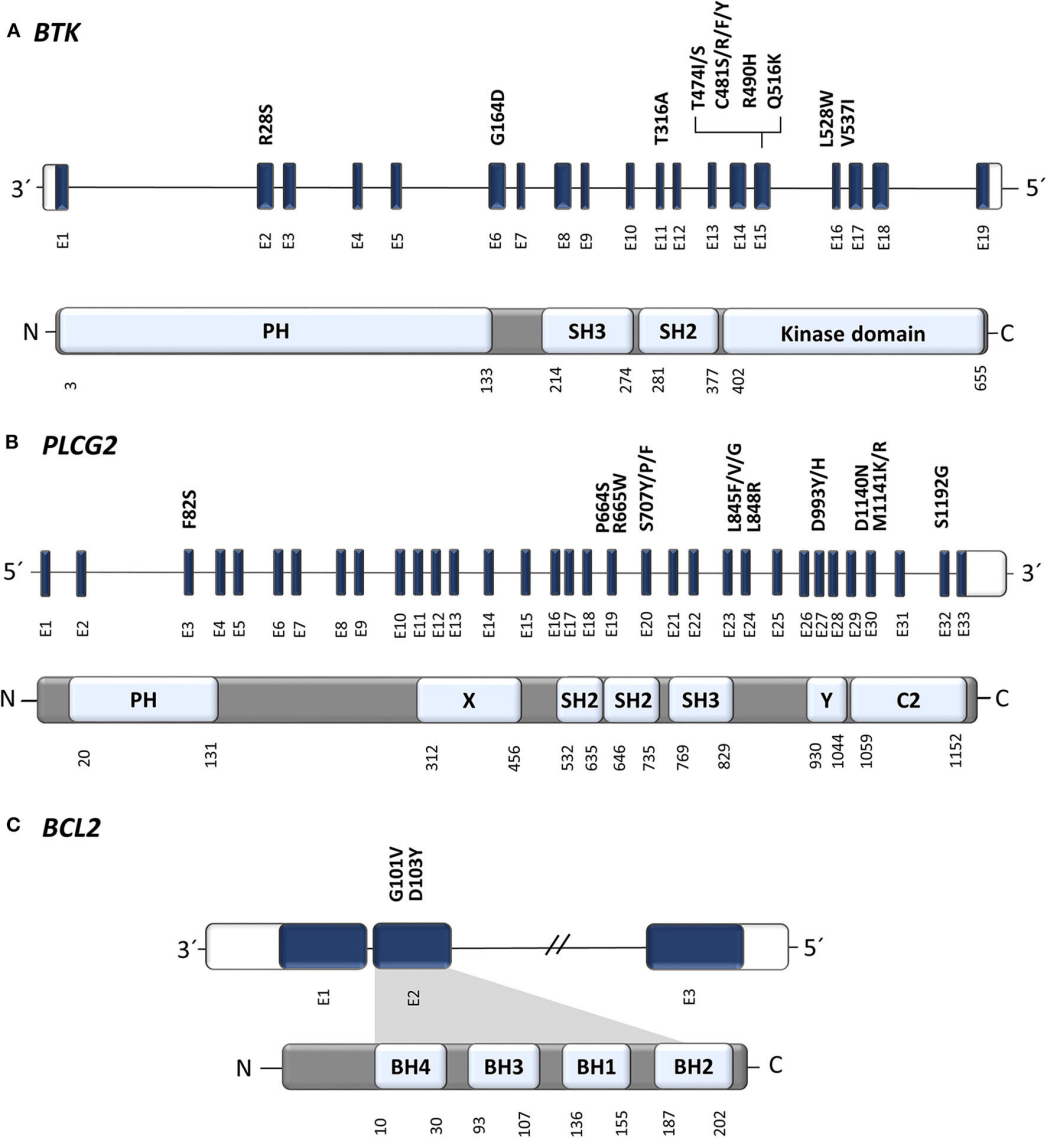
Position of known resistance-associated mutations in **(A)** *BTK*, **(B)** *PLCG2*, and **(C)** *BCL2* genes. E, exon; PH, Pleckstrin homology domain; SH2/3, Src homology domain 2/3 (autoinhibitory domain); C2, Ca binding motive; X, catalytic domain; Y, catalytic domain; BH1-4, BCL-2 homology domain 1-4. Gene structure has been visualized according to a genome browser Ensembl (Ensembl release 99—January 2020, EMBL-EBI); protein domains and amino acid positions according UniProt Knowledgebase (2020_01 release—February 2020, UniProt Consortium).

So far, most information about resistance-associated mutations has been described for patients treated with ibrutinib, an oral agent inactivating Bruton's tyrosine kinase (BTK). This antiproliferative and proapoptotic agent, acting via formation of an irreversible covalent bond at the C481 position of BTK, inhibits both B-cell receptor (BCR) and NF-κB pathways [Figure 1; (5, 6)]. Ibrutinib has been shown to be highly effective not only in R/R CLL patients with del(17p) and/or *TP53* mutation or complex karyotype (1, 2) but also in previously untreated patients with/without poor-risk features (3, 4). Primary resistance with no initial response to ibrutinib has been observed rarely and its mechanism is not yet understood (7–9). Acquired secondary resistance to ibrutinib occurs in 8–13% of CLL cases who responded well to the treatment initiation (7, 8) and are most commonly caused by the occurrence of resistant-associated mutations, as reported below (Figure 2).

**Figure 3:**
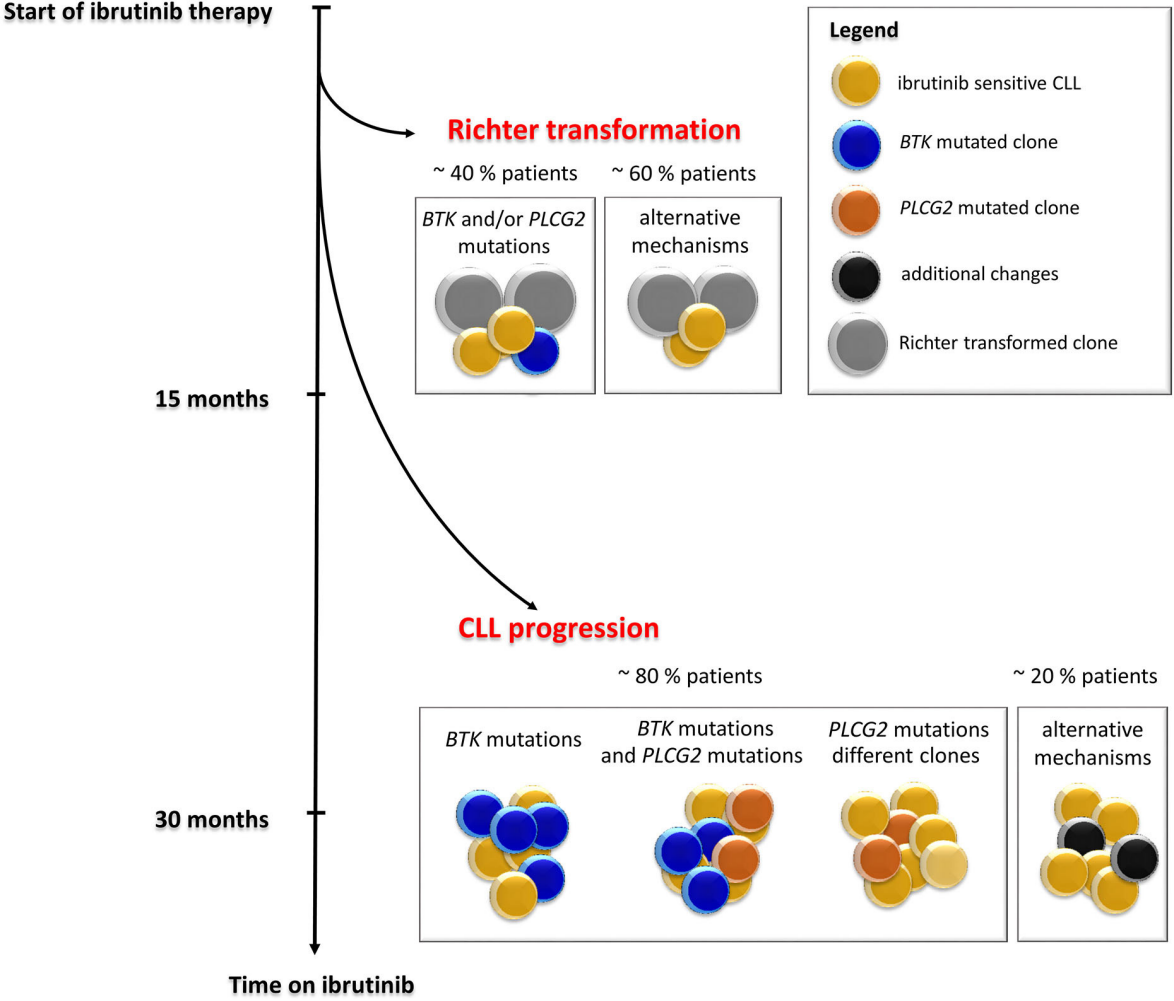
Timeline and frequency of occurrence of resistance-associated mutations emergence in CLL patients who progressed on ibrutinib treatment. Resistance-associated mutations in *BTK* and *PLCG2* genes usually develop between the second and fourth year of ibrutinib treatment. Mutated clones may occur at low allelic frequencies and may be detected several months before clinical relapse. Richter transformation is an early event that usually develops during the first 15 months of treatment with ibrutinib. Adapted from Ahn et al. (12).

In 2014, a study using whole-exome sequencing discovered acquired mutations within the *BTK* gene in 5/6 high-risk CLL patients relapsing on ibrutinib (10). A recent study on 30 CLL patients with residual lymphocytosis treated with ibrutinib for 3 years confirmed the presence of *BTK* mutations in 57% of CLL patients, and the presence of *BTK* mutations was associated with subsequent relapse (11). A number of studies have confirmed the presence of this mutation in CLL patients relapsing on ibrutinib and have shown that those mutations were not present prior to drug administration (12–14). The most common mutation (C481S) was found at the position of the binding site for ibrutinib thus reducing ibrutinib affinity for BTK (15, 16). More variants in the *BTK* gene, such as C481R, C481F, and C481Y, as well as less frequent variants at other gene positions (R28S, G164D, T316A, T474I/S, R490H, Q516K, L528W, and V537I), were revealed in later studies [Figure 2, Table 1; (12, 17–21)]. However, mutations outside of the kinase domain are rare (19). Functional characterization of these mutations has shown that the increase in ibrutinib dose is not sufficient to overcome the effect of the C481S/R/F/Y mutations (16). The *BTK* mutations usually develop between the second and fourth year of ibrutinib treatment (median 34.3 months, range 14–76.8 months) [Figure 3; (17)].

**Table 1:** Overview of selected studies describing the incidence of resistance-associated mutations in CLL patients treated with ibrutinib including selected studies describing resistance-associated mutations in patients with Richter transformation^#^.

**References**	**Number of patients**	**Genetic characteristics of patients**	**Number of previous treatment lines, median (min-max)**	**Frequency of patients with resistance-associated mutations**	***BTK*** **mutation**		***PLCG2*** **mutation**	
					**Variant(s)**	**VAF**	**Variant(s)**	**VAF**
Woyach et al. (10)	6	3/6 del(17p) + CK 1/6 del(17p) + trisomy 12 1/6 CK 1/6 del(11q)	4 (2–9)	100% (6/6)	C481S	17–60%	R665W S707Y L845F	8–38%
Burger et al. (23)	5	4/5 del(17p) + del(13q) 1/5 del(11q)	4 (1–6)	40% (2/5)	C481S	NA	S707F D993H M1141K/R	12–35%
Sharma et al. (19)^#^	1^*^	1/1 del(17p)	1	100% (1/1)	T316A	75%	0	0
Ahn et al. (12)^#^	10	10/10 del(17p)/mutation *TP53*	NA	80% (8/10)	C481S/R	2–78%	P664S R665W S707Y L845F 6 nt del	0.1–18%
Woyach et al. (13)	54	40% del(17p)^**^58% CK	3 (0–16)	89% (48/54)	C481S/R/F	0.2–100%	R665W S707Y/P/F L845F D993Y L845-846del	4–44%
Kadri et al. (14)^#^	9^***^	8/9 del(17p)	2 (1–4)	56% (5/9)	C481S/R T316A	3–90%	0	0
Quinquenel et al. (11)	30	15/30 mutation *TP53*4/30 mutated *IGHV*	2 (NA-NA)	57% (17/30)	C481S/Y/R/G	0.2–73%	R665W L845G C849R D993H	1–11%
Gángó et al. (21)^#^	20****	7/20 del(17p) 10/20 del(13q) 6/20 trisomy 12 3/20 del(11q)	3 (1–5)	40% (8/20)	R28S G164D R490H C481S/Y Q516K	2.7–27.3%	F82S R694H D993H S1192G	2.6–4.9%

*NA, not available; CK, complex karyotype; VAF, variant allele frequency*.

* *Mutation status of a patient with Richter transformation was described; resistance-associated mutation was found in peripheral blood, not in lymph nodes (19)*.

** *Patient characteristics for a complete set of 308 patients (13)*.

*** *6/9 patients with Richter transformation were included (14)*.

**** *3/20 patients with Richter transformation were included (21)*.

*PLCG2* gain-of-function mutations are the second most frequent mutations found in CLL patients who failed on the ibrutinib treatment (Figure 2). *PLCG2* is the gene encoding phospholipase Cγ2, the protein immediately downstream of BTK. These mutations mostly have an activating effect resulting in continuous BCR signaling independently on BTK activation (10, 22). *PLCG2* mutation hotspots are located in several domains of the gene and often co-occur with hotspot *BTK* mutations (11–13, 21, 23). Moreover, different *PLCG2* mutations are usually found in multiple subclones with low allelic burden (12, 13, 17). A recent study confirmed *PLCG2* mutations in 13% of ibrutinib treated patients with residual lymphocytosis (11). However, the exact contribution of the *PLCG2* to the clinical resistance of CLL patients remains not fully understood (22). Similarly, as *BTK* mutations, also *PLCG2* mutations usually occur between the second and fourth year on ibrutinib (median 35.1 months, range 17.4–64.6 months) [Figure 3; (17)].

Although mutations in *BTK* and *PLCG2* genes are detected in ~80% of CLL patients who failed on ibrutinib (10, 12, 13), for 20% of patients, ibrutinib resistance-associated mutations remain unknown (13, 14, 23). These data further support the presence of alternative mechanisms of drug resistance other than *BTK/PLCG2* mutations in a subset of patients who are still under investigation. To date, several candidate loci/mutations that may contribute to resistance have been described. These include del(8p) and *SF3B1, PCLO, EP300, MLL2*, and *EIF2A* mutations (10, 14, 23). More candidate genetic factors associated with resistance for ibrutinib-treated patients involve *BCL6* rearrangements, *MYC* gene abnormalities, del(17p), del(18p), 2p gain, *XPO1* overexpression, complex karyotype, epigenetic changes, and changes in the cell microenvironment (1, 14, 18, 24). However, it remains unclear whether these aberrations contribute causally to clinical resistance.

## *BTK* and/or *PLCG2* Mutations Clonality

Studies of follow-up samples revealed that mutations within the *BTK* and *PLCG2* genes are often present several months before clinical relapse is observed [Figure 3; (11–13)]. Those resistance-associated mutations were detected as early as 9.3 months prior to clinical progression (13). Moreover, Ahn et al. revealed that the resistance-associated mutations may be detected using highly sensitive approaches 15 months prior to disease progression (12).

The same *BTK* mutations have also been reported for CLL patients who experienced Richter transformation on ibrutinib treatment. In this group of patients, the resistance-associated mutations appeared in a smaller portion of patients (~40%) earlier, mostly within 15 months after ibrutinib treatment initiation [Figure 3; (13, 14)].

Nearly half of the patients who progress on ibrutinib carry *BTK* mutations in minor subclones with low variant allele frequencies (VAF) below 10% (12, 13). Nevertheless, cases with variants >80% VAF were also no exception (25). Clonal development and subclonal heterogeneity of resistance-associated mutations, i.e., the detection of multiple independent subclones carrying different variants of resistance-associated mutations with distinct growth rates, are also frequently observed in ibrutinib-treated patients (12, 23). In particular, it applies to *PLCG2* gene variants with lower VAF. However, the precise mechanism by which *BTK* and/or *PLCG2* mutations drive clinical resistance when present at such low allelic frequencies has not been elucidated yet.

In the recent study of Gángó et al., an association between reduction or even elimination of *TP53* mutated clones and the presence of *BTK* mutations has been reported (21). Vice versa, in patients with persisting *TP53* mutated subclones, no *BTK* mutations were detected in this study. The authors speculate that a longer duration of ibrutinib treatment could create conditions for the survival of subclones with *BTK* mutations by eliminating subclones with *TP53* mutations or that the elimination of *TP53* subclones enables the expansion of subclones harboring *BTK* mutations (21). The loss of preexisting mutations in *TP53* and *BIRC3* genes in patients who gained *BTK* mutations was also observed in another recent study (20). The relationship between *TP53* and *BTK* mutations should be confirmed on a larger patient cohort.

Another study showed that resistant mutated subclones and disease progression may occur only in specific compartments (13, 14). For some patients, Woyach et al. reported the presence of resistance-associated mutations only in lymph nodes without a presence in a corresponding peripheral blood sample (13). For these patients, the disease progression was also observed only in the lymph nodes. Therefore, it is necessary to choose the right collection material for the analysis of resistance-associated mutations, and even a negative result from peripheral blood may not mean that the patient does not have a subclone with a resistance-associated mutation in another location (25).

## Resistance-Associated Mutations in Idelalisib Treatment

Idelalisib is a phosphatidylinositol 3-kinase δ isoform (PI3Kδ) inhibitor, a member of the BCR inhibitor family, which not only inhibits PI3K signaling but also selectively induces apoptosis in CLL cells [Figure 1; (26)]. Idelalisib was approved for the treatment of patients with R/R CLL and high-risk patients with *TP53* disruption (27, 28). Regardless of its clinical efficacy, disease progression during idelalisib treatment is observed in some CLL patients (29–31).

Despite intensive research, the biological mechanism of the disease progression in idelalisib-treated patients remains unknown and no resistance-associated mutations in specific gene(s) or signaling pathway alterations have been found so far (29–31). A whole-exome sequencing study in a small cohort of 13 CLL patients who progressed on idelalisib treatment revealed that no mutations occurred in the PI3K signaling pathway or in any related signaling pathway (31).

These results indicate that there is probably no single pathway or specific mutation associated with idelalisib resistance thus deserving future investigations. A recent study investigating *in vivo* mouse models of resistance to PI3K inhibitors identified upregulation of genes from the integrin receptor complex as a possible mechanism of resistance (30). This suggests that resistance to idelalisib may be more likely mediated by dysregulation of survival signaling rather than by recurrent mutations. Nevertheless, it remains to be determined to what extent this mechanism of resistance plays a role in human CLL.

## Resistance-Associated Mutations in Venetoclax Treatment

Venetoclax is an oral BH3 mimetic and highly selective inhibitor of the BCL-2 antiapoptotic protein, capable of restoring apoptosis tumor cells with high overall response rates as well as in heavily pretreated, high-risk CLL patients. Venetoclax is primarily available for CLL patients with *TP53* disruption, for patients who failed on ibrutinib or were not suitable for the treatment with BCR signaling inhibitors, as well as for patients refractory to chemoimmunotherapy (32–34).

There is already growing evidence about the role of acquired mutations leading to the progression and failure of venetoclax. A recent study reported G101V mutation in the *BCL2* gene in 7 of 15 (47%) CLL patients progressing on venetoclax (35). Functional analysis demonstrated that G101V mutation disrupts the bond of venetoclax to BCL-2, therefore preventing the drug from competing with proapoptotic molecules to bind with BCL-2. The G101V mutation was absent at baseline, first detected 19–42 months after the initiation of venetoclax treatment and 25 months prior to clinical relapse, and persisted in five of seven patients for more than 6 months after the discontinuation of venetoclax therapy (35). The G101V mutation showed a high variability in VAF ranging from 1.4 to 70% (35).

Another recent study confirmed G101V in three of four CLL patients treated with venetoclax and found a second *BCL2* variant, D103Y, also within the BH3-binding site (36). The D103Y was acquired 39 months after the initiation of venetoclax therapy, at first with a low VAF of 7%, increasing to 18% over the next 5 months of venetoclax treatment in the reported patient. At a later date, this patient became positive also for G101V mutation with 14% VAF. NGS analysis showed that these two mutations exist as independent subclones with different growth dynamics, and they were not detected in the control group of 546 CLL patients not treated with venetoclax (36).

A whole-exome sequencing study in a small cohort of eight patients with del(17p) progressing on venetoclax identified a number of candidate resistance-associated aberrations, such as homozygous deletions of *CDKN2A/B* resulting in the loss of cell cycle control in three patients and mutations in the antiproliferative *BTG1* gene in two patients (37). Analysis of pretreatment samples revealed that these aberrations developed after the treatment initiation. In this study, the spectrum of other mutations in CLL-associated genes has been identified, such as *BRAF, NOTCH1, RB1, SF3B1*, and *TP53* mutations. Nevertheless, their causal relationship to resistance has not yet been established.

## Current Developments to Circumvent Failure on Novel Agents

Current studies have provided evidence that the early and sensitive detection of *BTK, PLCG2*, and *BCL2* mutations may be predictive of an impending relapse, as they occur several months before clinical relapse (35, 38). Therefore, the emergence of the resistance-associated mutations in patients receiving long-term treatment with BCR and BCL-2 inhibitors should be tested at regular intervals using highly sensitive ultradeep NGS approaches. As the resistance-associated mutations occur often at low VAF, it is necessary to standardize sequencing parameters in order to minimize the probability of false-positive and false-negative results (39–42).

In the case of a positive result for *BTK, PLCG2*, and *BCL2* mutations, the treatment should not be discontinued but rather different appropriate inhibitors or alternative combination therapies should be considered (43, 44). Therefore, understanding the nature of disease progression and emergence of clinical resistance has important implications, especially in the development of new treatment strategies that would help overcome resistance before it develops or as it emerges (43).

**Figure 4:**
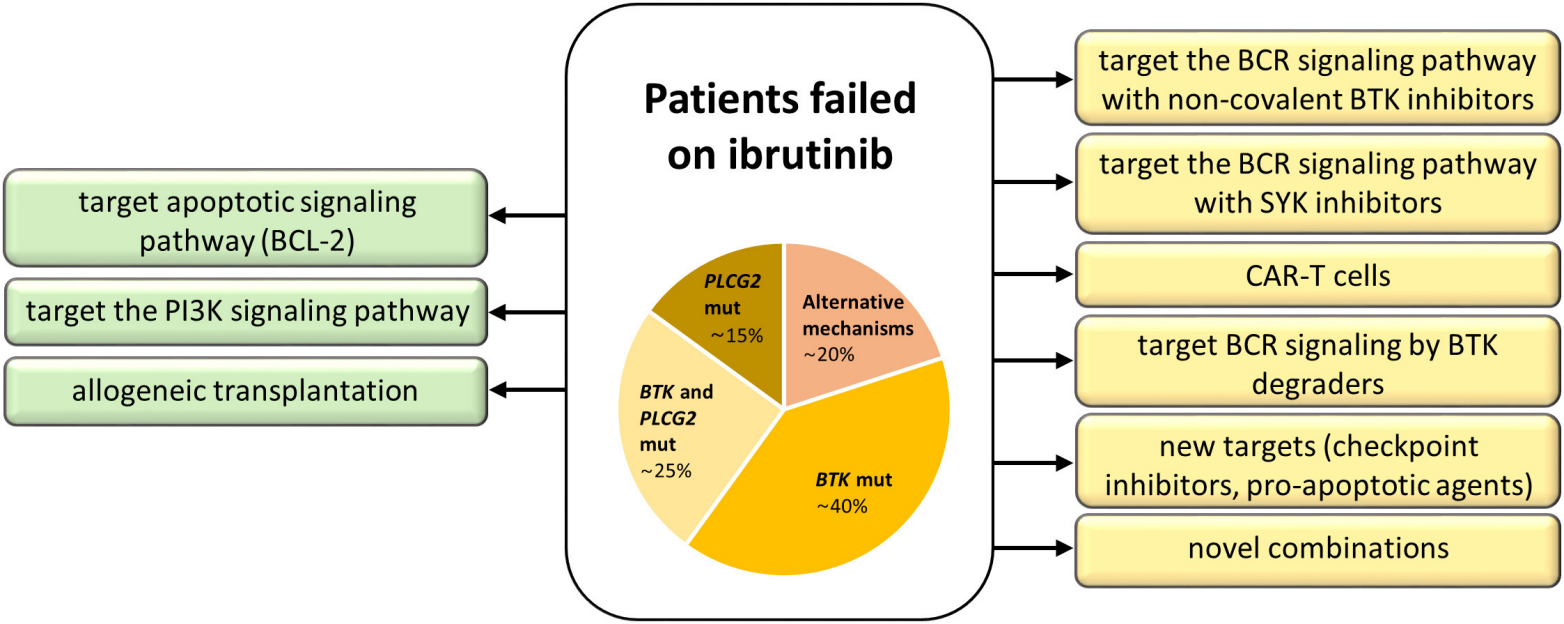
Treatment strategies for patients with acquired resistance to ibrutinib. Approved treatment options are on the left (green), experimental ones on the right (yellow).

**Figure 5:**
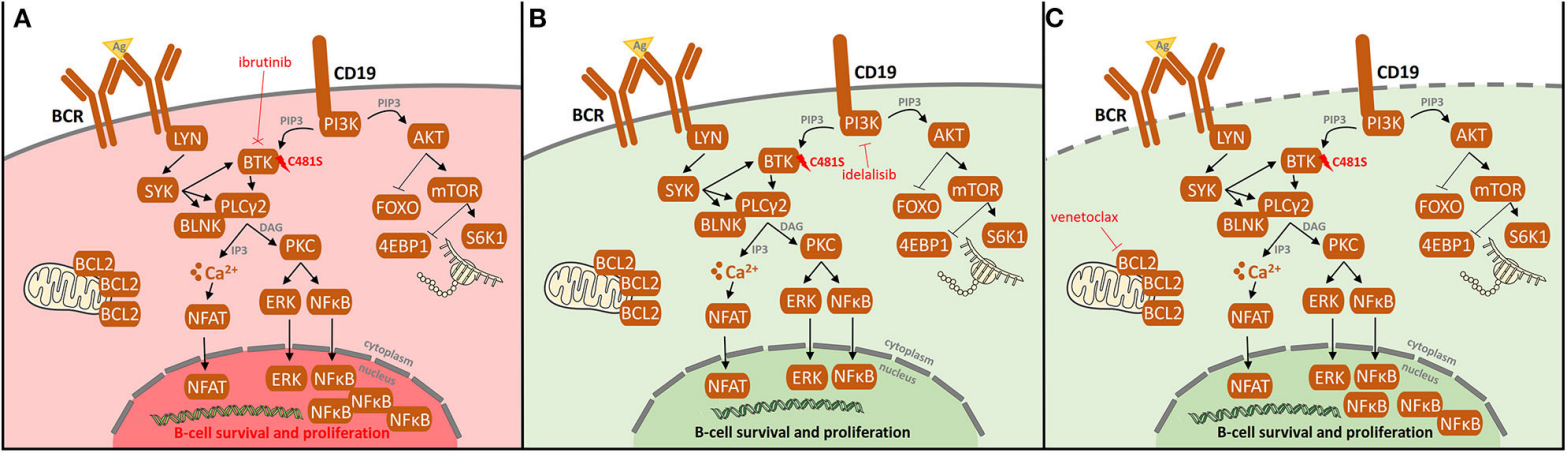
Resistance-associated *BTK* (C481) mutation and possibilities of its overcoming in ibrutinib-treated CLL patients. **(A)** After the occurrence of *BTK* (C481) mutation, ibrutinib does not bind to BTK causing the loss of its therapeutic effect leading to BCR and NF-kB pathway activation, both signaling pathways pivotal for maintenance and proliferation of CLL cells. Additionally, activating mutations in *PLCG2* gene may result in continuous BCR signaling independently on BTK activation. To overcome the resistance by mutations in *BTK*/*PLCG2* genes, other pathways may be targeted. **(B)** Idelalisib targets phosphatidylinositol 3-kinase (PI3K), resulting in the downregulation of PI3K/mTOR signaling pathway, irrespectively on the *BTK* mutation status. **(C)** Venetoclax targets BCL-2, thus leading to the apoptosis of CLL cells. As the previous treatment with BCR inhibitors results in an increased dependence on BCL-2 expression together with the fact that venetoclax complements ibrutinib-mediated apoptosis, a deep therapeutic effect on venetoclax is achieved.

There are currently several options, clinically proven as well as in experimental settings, how to circumvent failure on ibrutinib and other novel agents (Figure 4). The most promising is to target alternative molecules or signaling pathways involved in the survival of malignant cells (Figure 5). Another therapeutic intervention could be the use of different combination therapies to reverse relapse (43). Very promising is the combination of ibrutinib with anti-CD20 antibodies (28). In addition, the combination of ibrutinib with venetoclax represents a great potential in the treatment of CLL, as there is increasing evidence that CLL cells previously treated with BCR inhibitors show an increased dependence on BCL-2 expression (45, 46). This is further supported by the observation of the changes in BCL-2 family protein levels in CLL cells when treated with ibrutinib (45) and acalabrutinib (47). Additionally, venetoclax complements ibrutinib and acalabrutinib–mediated apoptosis in CLL cells (45, 47). The synergic effect of both drugs acting by different mechanisms has led to a deep therapeutic effect in CLL (45, 46, 48); however, the exact mechanism of their interaction in CLL should be further elucidated.

## Next-Generation Inhibitors and Experimental Treatment Options

New covalent BTK inhibitors with high selectivity to BTK, such as acalabrutinib (ACP-196), zanubrutinib (BGB-3111), and tirabrutinib (ONO/GS-4059), have high therapeutic potential [Figure 1; (49–51)]. However, like ibrutinib, they covalently bind to BTK and are, therefore, not suitable for the treatment of patients with resistance-associated *BTK* hotspot mutation (C481S/R/F/Y). Compared to ibrutinib, the recently approved acalabrutinib (52) shows a more acceptable safety profile and modulation of BCL-2 family proteins contributing to cell death induced by venetoclax after acalabrutinib treatment in CLL (47). Similarly to ibrutinib, acquired resistance to acalabrutinib was mainly associated with *BTK* mutations (53). Zanubrutinib is the next potent and highly selective inhibitor of BTK, currently approved for mantle cell lymphoma treatment and tested in clinical trials for CLL (52). In four CLL patients progressing on zanubrutinib treatment, Handunnetti et al. identified *BTK* C481 and L528W mutations, both of them absent prior to zanubrutinib treatment (54).

Importantly, a new generation of non-covalent BTK inhibitors is currently being tested such as fenebrutinib (GDC-0853), ARQ-531, LOXO-305, and vecabrutinib (SNS-062) (51, 52, 55). The first clinical studies with fenebrutinib have shown that non-covalent selective inhibition of BTK may be effective in CLL patients with acquired resistance to ibrutinib therapy (56). But *in vitro* mutagenesis of the *BTK* gene has shown that mutations in the kinase domain (L512M, E513G, F517L, and L547P) reduce the effect of these new non-covalent BTK inhibitors (57). Also, ARQ-531, an ATP-competitive non-covalent reversible inhibitor, is able to overcome *BTK* (C481S) and *PLCG2* (R665W, S707P, S707F, R742P, and L845fs) mutations as shown in animal models (58). Similarly, the reversible BTK inhibitor LOXO-305 shows great potential in overcoming acquired resistance to irreversible BTK inhibitors in preclinical CLL models; phase 1 clinical trial of LOXO-305 is currently ongoing (59).

Among experimental treatment options in patients who failed on ibrutinib belong SYK (Spleen tyrosine kinase) inhibitors, chimeric antigen receptor T (CAR-T) cell therapy, anti-PD-1 treatment, and BTK degraders (Figure 4). SYK is a kinase upstream of both BTK and PI3Kδ in the BCR signaling pathway. The SYK inhibitor entospletinib shows a clinical activity for R/R CLL patients who have relapsed on BTK or PI3Kδ inhibitors, even in the presence of *BTK* and *PLCG2* mutations (44). CAR-T cell therapy (60) represents another treatment opportunity for CLL patients failing BCR or BCL-2 inhibitor therapies, currently available only in clinical trials (52). Further, response to PD-1 blockade with pembrolizumab has been observed in patients with Richter transformation who had progression after prior therapy with ibrutinib, but not in R/R CLL patients (61). Another emerging approach evidenced to have an effect on both wild-type and mutated BTK (C481S) in preclinical studies is the novel agent BTK degrader, MT-802, causing ubiquitination of BTK, and subsequent degradation through proteasome (62). A recent study on animal models has shown the possibility to overcome resistance to ibrutinib by preventing FOXO3a nuclear export and PI3K/AKT activation (63). These promising data and the development of new approaches to overcome acquired resistance to current BCR and BCL-2 inhibitors show great hope not only for CLL patients.

## Conclusion

With increasing experience in the treatment with novel agents, it has become extremely important to (i) detect genetic aberrations associated with resistance and progression and (ii) understand the mechanisms of resistance development and disease progression in patients treated with these agents. Resistance-associated mutations in *BTK, PLCG2*, and *BCL2* have the potential to be used as a biomarker for future relapse or disease progression and hence their detection could facilitate early therapeutic intervention therapy to prevent the relapse. In addition, understanding the mechanism of resistance may also help find a way on how to prevent resistance before it develops or to overcome as it emerges.

In the near future, we can expect an increase in the number of patients indicated for inhibitor therapy due to the excellent performance of these agents, not only in CLL. Since patients are treated for longer with these drugs, it will certainly be reflected in the increased incidence of disease progression and failure on those agents; hence, the challenge of managing resistance and identifying which patients are at risk for relapse is of the highest importance. There is a long way to learn on how to manage a resistant disease that we will encounter more and more often in the era of treatment with novel targeted agents.

## Data Availability Statement

All datasets generated for this study are included in the article.

## Author Contributions

LS and EK wrote the manuscript. LS and AP prepared the figures and revisions. TP, PT, and AP revised the manuscript critically. EK contributed to conceptualization, study design, writing, and editing. All authors contributed to the article and approved the submitted version.

## Conflict of Interest

The authors declare that the research was conducted in the absence of any commercial or financial relationships that could be construed as a potential conflict of interest.
